# The First 24 Hours of Hospital Admission: Early Opportunity for Pharmacotherapy Optimization in Elderly Patients with Chronic Kidney Disease

**DOI:** 10.3390/pharmacy14060130

**Published:** 2026-09-04

**Authors:** Ana Mulej, Ivana Marinović, Iva Matković, Vesna Bačić Vrca, Luka Torić, Dino Kasumović, Matija Crnogorac, Ivica Horvatić, Ivana Samardžić

**Affiliations:** 1 Hospital Pharmacy, Clinical Hospital Center of Rijeka, 51000 Rijeka, Croatia; ana.mulej@kbc-rijeka.hr; 2 Department of Clinical Pharmacy, University Hospital Dubrava, 10000 Zagreb, Croatia; imatkovic1@kbd.hr (I.M.); isamardzi@kbd.hr (I.S.); 3 Faculty of Pharmacy and Biochemistry, University of Zagreb, 10000 Zagreb, Croatia; bacicvrca@gmail.com (V.B.V.); ivica.horvatic@yahoo.com (I.H.); 4 Department of Nephrology and Dialysis, University Hospital Dubrava, 10000 Zagreb, Croatia; ltoric@kbd.hr (L.T.); dkasumovic@kbd.hr (D.K.); mcrnogorac@kbd.hr (M.C.); 5 School of Medicine, University of Zagreb, 10000 Zagreb, Croatia

**Keywords:** chronic kidney disease, elderly patients, polypharmacy, potentially inappropriate medications, drug–drug interactions, renal-risk drugs, clinical pharmacist

## Abstract

Chronic kidney disease (CKD) affects more than 10% of the global population and represents one of the leading causes of mortality. This study aimed to assess the pharmacotherapy of hospitalized patients aged ≥ 65 years with CKD, focusing on the prevalence of polypharmacy, potentially inappropriate medications (PIMs), potentially clinically significant drug–drug interactions (DDIs), the use of renal-risk drugs (RRDs). A prospective observational study was conducted at the Department of Nephrology and Dialysis of University Hospital Dubrava, including 100 participants. A clinical pharmacist obtained the Best Possible Medication History (BPMH) within 24 h of hospital admission. A total of 1324 comorbidities were recorded (median 12 per patient), along with 985 prescribed medications. Polypharmacy (5–9 drugs) was observed in 43% of patients, while excessive polypharmacy (≥10 drugs) was present in 48%. A total of 258 PIMs were identified, with 89% of patients having at least one PIM (mean 2.6 per patient). Nearly 94% of patients had significantly impaired renal function (G3–G5), and 82% were exposed to inappropriately prescribed RRDs. Contraindicated medications based on renal function were identified in 62% of patients, while 52% had at least one medication prescribed at an unadjusted dose. A total of 1097 potentially clinically significant DDIs were identified, with a mean of 10.9 interactions per patient. The results indicate a high prevalence of polypharmacy, PIMs, potentially clinically significant DDIs and inappropriately prescribed RRDs among older hospitalized patients with CKD, increasing the risk of adverse events and unfavorable treatment outcomes. The findings highlight the importance of the 24 h period following hospital admission as an early opportunity for identifying medication-related problems and optimizing pharmacotherapy in highly vulnerable patients, such as elderly patients with CKD. The study also emphasizes the importance of early involvement of a clinical pharmacist in the pharmacotherapy review and obtaining the BPMH.

## 1. Introduction

CKD represents a significant public health problem and is associated with numerous complications affecting multiple organ systems [1,2]. It is estimated that over 850 million people worldwide are living with CKD, which is responsible for more than 3 million deaths annually, with a continuing upward trend [3]. Due to nonspecific symptoms and the kidneys’ ability to compensate for loss of function, the disease often remains unrecognized until advanced stages [2]. Early detection and appropriate management, including dose adjustment according to the degree of renal impairment and the removal of reversible factors, are essential for slowing disease progression and improving treatment outcomes [2].

Older adults represent a large and growing proportion of CKD patients. The prevalence of CKD increased sharply with age, affecting around 47% of individuals aged 75 and over [4]. Population aging is the leading demographic trend of the 21st century. Europe is aging rapidly, and people aged 65 and over account for more than one-fifth of the European Union’s population (22.4% in 2025). According to the latest Eurostat data, the median age of the population in the European Union has reached 44.9 years, meaning that half the population is older and half is younger than that age [5]. Medication-related problems are most pronounced in elderly people due to a number of physiological changes that may affect the pharmacokinetics and pharmacodynamics of the drug, frailty, cognitive impairment and complex health conditions. As kidney function declines, the burden of comorbidities increases, leading to complex medication regimens and a high prevalence of polypharmacy [6].

Polypharmacy in patients with CKD has been associated with increased mortality, accelerated decline in kidney function, reduced medication adherence, potentially inappropriate medications (PIMs), drug–drug interactions (DDIs), and poorer quality of life [7,8]. PIMs represent an important clinical challenge, particularly in older patients in whom the risks of use often outweigh the expected benefits [9,10]. DDIs may result in toxicity, reduced therapeutic efficacy, or serious adverse events. DDIs account for a substantial proportion of hospitalizations, further emphasizing the need for systematic medication review [11]. A particular pharmacotherapy problem in patients with CKD is the use of renal-risk drugs (RRDs) that require dose adjustment or are contraindicated due to reduced renal function [12]. The risk of nephrotoxicity is further increased in the presence of comorbidities, reduced glomerular filtration, and the concomitant use of multiple medications. Nephrotoxic drugs are a significant cause of acute kidney injury, especially in hospitalized elderly patients [13,14].

Given the complexity of managing older patients with CKD, a multidisciplinary approach involving physicians, clinical pharmacists, and other healthcare professionals is essential. The first 24 h following hospital admission represent one of the most critical points in patient care. Therefore, at this point it is important to review and optimize the complex medication regimens of patients with CKD. It is necessary to obtain the Best Possible Medication History (BPMH) and to conduct structured medication review, using explicit prescribing criteria to discontinue high-risk medications [15]. The systematic evaluation of pharmacotherapy, dose adjustment according to renal function, and timely identification of PIMs and DDIs are key steps in reducing the risk of adverse events and improving the safety and effectiveness of treatment in this vulnerable population [2]. KDIGO recommends assessing medication appropriateness and their number, dose, and potential interactions in patients with CKD [16]. Therefore, this study assessed polypharmacy, PIMs, potentially clinically significant DDIs and inappropriately prescribed RRDs, using the BPMH as a tool for pharmacotherapy analysis.

## 2. Materials and Methods

### 2.1. Setting and Participants

A prospective observational study was conducted at the Department of Nephrology and Dialysis of Clinical Hospital Dubrava. University Hospital Dubrava is a tertiary care institution with 600 beds, whose emergency department provides healthcare services to a population of approximately 350,000 inhabitants. The study was carried out between November 2023 and February 2025. The study protocol was approved by the Ethics Committee of University Hospital Dubrava and the Ethics Committee for Experimental Work of the Faculty of Pharmacy and Biochemistry. The study included hospitalized patients aged ≥ 65 years with CKD admitted to the Department of Nephrology and Dialysis, University Hospital Dubrava. Eligible participants were those whose clinical condition allowed the collection of a BPMH. For patients unable to provide reliable medication history due to their clinical condition, the BPMH was obtained from family members and/or caregivers. Patients for whom it was not possible to obtain a complete BPMH were excluded from the analysis. A clinical pharmacist obtained the BPMH within the first 24 h of admission. All participants or their legally authorized representatives provided written informed consent prior to enrollment.

### 2.2. Data Collection

A clinical pharmacist obtained the BPMH through an interview with the patient. The BPMH form included the following data: age, gender, body weight, social status, level of education, reason for hospital admission, comorbidities, previous hospitalizations (within one year prior to the current admission), and details of medications the patient was taking prior to hospital admission. These data included the number of medications, their names, doses, and routes of administration, and covered prescription drugs, over-the-counter medications, dietary supplements, and herbal products. Lifestyle habits, including alcohol consumption and smoking status, as well as known allergies, were recorded. Demographic and clinical data were also collected. All available sources used to obtain the BPMH were documented, such as previous hospital records, discharge summaries, laboratory data, examination of medication vials, review of the patient’s medication list, and interviews with caregivers or family members.

### 2.3. Outcomes Measures

Data collected using the BPMH were statistically analyzed to determine the prevalence and patterns of polypharmacy, as well as the prevalence of PIMs and the use of inappropriately prescribed RRDs in patients with CKD. All medications were classified according to the Anatomical Therapeutic Chemical (ATC) classification system. Based on the number of medications used, participants were categorized into three groups: those using less than five medications, patients with polypharmacy (5–9 medications), and patients with excessive polypharmacy (≥10 medications). PIMs were identified using the EU(7)-PIM list, a standardized tool developed for use in European countries comprising 282 active substances across 34 therapeutic groups. Data on renal function were obtained at hospital admission. Estimated glomerular filtration rate (eGFR) and creatinine values were available in the laboratory test results, whereas creatinine clearance had to be calculated. Creatinine clearance was calculated using the Cockroft–Gault equation. eGFR was calculated using the CKD-EPI equation. Based on eGFR values and KDIGO classification, patients were categorized into the following stages: G1 (≥90 mL/min/1.73 m^2^), G2 (60–89 mL/min/1.73 m^2^), G3a (45–59 mL/min/1.73 m^2^), G3b (30–44 mL/min/1.73 m^2^), G4 (15–29 mL/min/1.73 m^2^), and G5 (<15 mL/min/1.73 m^2^). The analysis of RRDs, including medications contraindicated in renal impairment and those requiring dose adjustment according to renal function, was performed using BPMH data and the corresponding official Summaries of Product Characteristics (SmPCs). SmPCs are available in the Medicinal Products Database of Croatian Agency for Medicinal Products and Medical Devices [17]. Inappropriately prescribed RRDs were defined as those with unadjusted doses or those contraindicated according to the degree of renal impairment. Dose adjustment was performed based on eGFR or creatinine clearance in accordance with SmPC recommendations. DDI analysis included all prescribed and over-the-counter medications recorded in the BPMH. DDIs were assessed using the Lexicomp^®^ database, which classifies potential interactions into five categories (A, B, C, D, and X) based on clinical significance. Potential DDIs classified as C, D, and X—defined as requiring monitoring, consideration of therapy modification, or contraindicated use—were considered clinically significant and included in further analysis.

### 2.4. Statistical Analysis

Data were analyzed using Microsoft Excel (version 16.0.14334.20848 64-bit). Descriptive statistics were used to summarize the demographic and clinical characteristics of the participants, as well as the prevalence of polypharmacy and the use of renal-risk medications in patients with impaired renal function. Continuous variables were presented as median and interquartile range, while categorical variables were expressed as frequencies and percentages.

## 3. Results

### 3.1. Demographic Characteristics

A total of 100 participants aged ≥65 years with CKD were included in the study. The median age was 76 years (71–82). The median number of comorbidities was 12 (IQR 9–15), and the median number of medications recorded in the BPMH was nine (IQR 7–13). According to the KDIGO classification, the majority of patients were classified in stages G5 (48%) and G4 (29%) of renal impairment. Detailed patient characteristics are presented in Table 1.

### 3.2. Polypharmacy

In 9 out of 100 participants, fewer than five medications were recorded (Table 2). Polypharmacy (5–9 medications) was identified in 43 participants (43%), while excessive polypharmacy (≥10 medications) was observed in 48 participants (48%). The median number of medications per patient in the BPMH was nine (IQR 7–13). The most represented ATC groups were drugs acting on the cardiovascular system (C), the alimentary tract and metabolism (A), and the nervous system (N). Within the cardiovascular group, diuretics were the most frequently prescribed medications (N = 94). The prevalence and distribution of polypharmacy, potentially clinically significant DDIs, PIMs, and inappropriately prescribed RRDs in the study population is presented in Table 3.

### 3.3. Potentially Inappropriate Medications

A total of 258 PIMs were identified in the BPMH using the EU(7)-PIM list. At least one PIM was present in 89 participants (Table 3). The mean number of PIMs per participant was 2.6, with a maximum of 7 PIMs identified in a single participant. A total of 48 different PIMs were identified. ATC group C was the most frequently represented therapeutic category, while pantoprazole was the most frequently identified individual PIM (Table 4).

### 3.4. Inappropriately Prescribed Renal-Risk Drugs

A total of 192 inappropriately prescribed RRDs were identified in patients with impaired renal function based on BPMH data. Of these, 67 required dose adjustment, while 125 were contraindicated according to the level of renal impairment. Dose adjustment was most commonly required in patients with a creatinine clearance of 30–60 mL/min, whereas contraindications were predominantly identified in patients with a creatinine clearance below 30 mL/min. Inappropriately prescribed RRDs (unadjusted doses and/or contraindicated drugs) were identified in 82% of patients (Table 3). Contraindicated medications were present in 62% of patients, while 52% had at least one medication with an unadjusted dose. The most frequently identified inappropriately prescribed RRDs included perindopril (including fixed-dose combinations), acetylsalicylic acid, moxonidine, tramadol (also in fixed-dose combinations), and lercanidipine. A total of 35 medications required dose adjustment, most commonly moxonidine, metoclopramide, and perindopril. Additionally, 40 medications contraindicated in patients with CKD were identified, most commonly acetylsalicylic acid, perindopril (including fixed-dose combinations), tramadol, and lercanidipine (Table 5).

### 3.5. Potentially Clinically Significant Drug–Drug Interactions

A total of 1097 potentially clinically significant DDIs were identified through BPMH analysis. Of these, 984 interactions were classified as category C and required enhanced patient monitoring (89.7%), 100 interactions required therapeutic intervention (category D; 9.1%), and 13 interactions were contraindicated (category X; 1.2%). The mean number of potentially clinically significant DDIs per patient was 10.9. At least one category C interaction was identified in 94 patients (94%), while 54 patients (54%) had at least one category D interaction. Category X interactions were identified in 10 patients (10%). The most common potentially clinically significant DDIs are presented in Table 6.

## 4. Discussion

The first 24 h following hospital admission represents an early opportunity to identify medication-related problems and optimize pharmacotherapy. A delay in obtaining BPMH is associated with an increased risk of medication errors and can jeopardize patient safety [18]. This study assessed polypharmacy, PIMs, potentially clinically significant DDIs and inappropriately prescribed RRDs in hospitalized elderly patients with CKD within 24 h of hospitalization. The study determined their high prevalence and indicated the therapy complexity in elderly patients with CKD. Optimizing therapy is challenging in elderly people, especially for patients 75 and older. It is well known that patients over the age of 75 face specific health challenges. Our study included patients aged 65 and older and the percentage of patients with CKD aged 75 and over was 59%. The median number of comorbidities in our study was 12 (9–15). Hypertension (83%), anemia (64%), disorder of fluid, electrolyte and acid–base balance (53%), and type 2 diabetes (45%) were the most common comorbidities in the study population. Hypertension and diabetes are leading causes of CKD, but they can also be consequences of it, thereby creating a risky and bidirectional vicious cycle. Anemia and electrolyte imbalances are common and serious consequences of CKD, manifesting primarily through reduced erythropoietin production, disrupted potassium balance, and the retention of phosphate and water. As the kidneys lose function, the body struggles to eliminate excess substances and loses the ability to maintain a normal blood profile and chemical balance.

In this study, a high prevalence of polypharmacy (91%) was observed, with excessive polypharmacy (≥10 medications) being more common than polypharmacy which included the use of 5–9 medications (48% vs. 43%). The results are consistent with recent systematic reviews reporting polypharmacy prevalence estimates ranging from approximately 69% to over 80% among patients with CKD [9,19,20,21].

We used the EU(7)-PIM list for PIM identification, as it was developed for the European healthcare system. Application of the EU(7)-PIM list identified 258 PIMs, with 89% of patients having at least one PIM in their medication regimen with an average 2,6 PIMs per patient. Previous reports also demonstrate a high proportion of potentially inappropriate prescribing among elderly patients with CKD [22,23,24]. In a retrospective study of elderly patients (≥65 years) with CKD treated in a nephrology department, the prevalence of PIMs was 91% according to the Beers criteria, 42% according to the STOPP criteria, and 70% according to the Medication Appropriateness Index (MAI) [24]. Similarly, a German study involving 375 older patients with CKD reported that between 43.2% and 79% of patients had at least one PIM, depending on the assessment criteria used. Patients receiving ten or more medications had a mean of 3.0 ± 1.7 PIMs [23]. Importantly, the clinical significance of PIM use extends beyond inappropriate prescribing itself, as accumulating evidence suggests that it may contribute to adverse renal and clinical outcomes. A large retrospective cohort study from Japan demonstrated that exposure to two or more PIMs was associated with a significant decline in eGFR of 20–30% and accelerated CKD progression [22]. Pantoprazole was the most frequently identified PIM in our study. Other studies also determined proton pump inhibitors (PPIs) as one of the most commonly inappropriately prescribed medications in older patients with CKD [24]. PPIs are considered as PIMs due to an increased risk of *C. difficile* infection and hip fracture. Although PPIs are widely prescribed, their long-term use is frequently not supported by an appropriate clinical indication or therapy duration and has been associated with avoidable medication-related risks [25,26,27]. Furthermore, it is important to highlight that some of the larger observational studies report higher rates of developing CKD in patients who take PPIs for a long time [28,29]. Patients should use PPIs only when necessary and this use should be regularly re-evaluated [16]. Discontinuing PPIs requires a gradual dose reduction and dose tapering according to indications and recommended protocols [30]. Deprescribing has emerged as an important strategy for optimizing pharmacotherapy. It is a patient-centered, evidence-based process of systematically identifying and discontinuing medications for which the potential harms outweigh the expected benefits or are no longer clinically indicated. This process requires an individualized approach that considers the balance of benefits and risks, multimorbidity, and patient preferences [31,32].

Most participants had advanced CKD, with 29% and 48% classified as KDIGO G4 and G5, respectively. This advanced stage of kidney disease markedly increases the risk of drug accumulation, toxicity, and medication-related adverse events, as many drugs are predominantly excreted via the kidneys. In our study, 82% of patients with impaired kidney function had at least one inappropriately prescribed RRD, either due to an inadequate dose or the use of a contraindicated drug. These findings indicate a higher prevalence of the inappropriate prescribing of RRDs than that reported in some previous studies (45%) [33]. This difference may be explained by the higher proportion of patients with advanced CKD (G4–G5) included in our cohort, who require more frequent dose adjustments and are more likely to receive medications contraindicated in severe renal impairment. Using the BPMH, we identified 35 medications requiring dose adjustment (52% patients) and 40 contraindicated medications (62% patients). With angiotensin-converting enzyme (ACE) inhibitors, problems related to dosing and use in impaired renal function are most often found. When creatinine clearance is below 60, dosage adjustment for ACE inhibitors is required, and ACE inhibitors were considered contraindicated if they were in fixed combinations with amlodipine and indapamide. An increase in creatinine levels of more than 30% from the start of pharmacotherapy treatment may also be a contraindication for the continuation of ACE inhibitor therapy. All ACE inhibitors are predominantly excreted via the kidneys, except for trandolapril, which is predominantly excreted via the liver > 70%. Similar findings have been reported in previous studies, emphasizing the importance of individualized dose adjustment and careful monitoring of kidney function, particularly when prescribing ACE inhibitors [34]. Failure to recognize RRDs may contribute to further deterioration of kidney function, hyperkalemia, hypotension, and acute kidney injury, highlighting the clinical importance of systematic medication review at hospital admission [16,35,36].

Multimorbidity and polypharmacy contributed to the high prevalence of potentially clinically significant DDIs in these patients. DDIs can complicate the course of treatment and compromise patient safety. We use Lexicomp as a tool for the identification of DDIs due to its high sensitivity and specificity (≥90%). A total of 1097 potentially clinically significant DDIs were detected, of which 89.7% were classified as category C, 9.1% as category D, and 1.2% as category X. The mean number of potentially clinically significant DDIs per patient (10.9) was even higher than the mean reported in previous studies evaluating potential DDIs among patients with CKD [37,38]. Interactions in category C had the potential to increase the risk of hypotension. The most common interacting agents were from ATC group C. Category D interactions most frequently involved combinations of beta-blockers and α2-receptor agonists, reflecting the widespread use of antihypertensive therapy in patients with advanced CKD. These combinations may increase the risk of rebound hypertension following abrupt discontinuation and exacerbate atrioventricular conduction disturbances. Category X interactions were mainly associated with increased risk of enhanced hypotensive effects and QTc interval prolongation, particularly in combinations involving diuretics and promazine. These interactions require particular attention because they generally warrant avoidance of the combination or prompt modification of therapy. Previous studies have shown that the occurrence of potentially clinically significant DDIs in CKD patients increases with older age, prolonged hospitalization, and a higher number of prescribed medications [37]. Managing interactions requires creating a model that delineates the respective interventions of physicians and pharmacists tailored to the care of patients with CKD. Insight into the distribution of interventions in DDI management models between physicians and pharmacists contributes to more efficient pharmaceutical care and visibility [39].

The study highlights the burden of comorbidities and the complexity of treatment in patients with CKD. The presence of certain drug classes is particularly notable among the observed therapeutic issues. Particular emphasis in the therapy assessment must be placed on antihypertensives, antidiabetics, NSAIDs, and PPIs, as these groups directly affect renal hemodynamics, accumulate metabolically due to reduced clearance, and carry an extremely high risk of toxicity and further acceleration of disease progression. Continuous patient education is necessary to avoid irrational use over-the-counter analgesics or PPIs. The introduction of any new medication presents a clinical challenge due to the risk of adverse interactions and requires strictly individualized dose adjustment based on the eGFR. Any dosage decision must be based on current, rather than old, laboratory values for renal function.

The management of therapy in patients with CKD has numerous obstacles. The type and frequency of medication-related problems, the length of hospital stay, and prior hospitalizations are the most important predictive factors for developing advanced care models that reduce readmission rates and improve the quality of life for patients with CKD. Furthermore, the latest KDIGO guidelines emphasize the importance of clinical pharmacists’ involvement in drug stewardship in patients with CKD. Medication review and medication reconciliation are essential for minimizing the occurrence of medication-related problems and enhancing patient safety. Frequent medication reviews may be needed in older adults with complex medication regimens compared with younger people with CKD [16]. The importance of the timely involvement of a clinical pharmacist in medication review for hospitalized older patients with CKD following emergency admission, as was the majority of our patients, should be particularly noted. The early involvement of a clinical pharmacist enables the timely recognition of medication-related problems, given their specific expertise in pharmacotherapy management. Their specific expertise includes identification of inappropriate drug selection, dosing errors, DDIs, adverse drug reactions, and adherence issues. The timely identification of medication-related problems can reduce treatment failure, adverse events, and enhance patient safety.

In addition, at hospital admission, the clinical pharmacist has a bridging role in pharmacotherapy transfer. A randomized controlled study conducted in Croatia on an elderly patient population showed a significant reduction in medication errors in the transfer of care from hospital to primary care. The care transfer model was led and organized by a hospital clinical pharmacist. Collaboration with primary health care involved community pharmacists and family medicine physicians [40]. The healthcare system should strive to implement standardized models of cooperation between physicians and community pharmacists [39,40].

This study had several limitations. The study was designed as an observational study. It was conducted in one center and included only inpatients at the time of admission. A multi-center design in future studies would include regional variations in clinical protocols and prescribing patterns and interventions among nephrologists and clinical pharmacists. Other limitations were the monitoring of only potentially clinically significant DDIs, a relatively small sample from a single center, and a predominance of patients with advanced CKD. Since CKD patients are highly susceptible to acute kidney injury and nosocomial infections during their stay, tracking DRPs continuously throughout the hospital stay would provide a more comprehensive insight into drug-related risks. It would also be useful to identify medication-related problems at discharge that require patient monitoring in an outpatient setting. The transition from inpatient to outpatient care is a high-risk period for medication errors. Identifying DRPs at discharge is crucial for creating effective handover protocols for primary care physicians and community pharmacists. Further research should distinguish the distribution of care within the multidisciplinary team in elderly patients with CKD in hospital and outpatient settings to prevent the fragmentation of care and to make it more efficient.

## 5. Conclusions

This study demonstrated the substantial complexity of pharmacotherapy in hospitalized elderly patients with CKD, identifying a high prevalence of polypharmacy, PIMs, potentially clinically significant DDIs and inappropriately prescribed RRDs. These findings also highlight the importance of implementing early and systematic strategies for the identification and management of medication-related problems, particularly in a vulnerable patient population.

A structured medication review led by a hospital clinical pharmacist, based on the BPMH, enables a comprehensive assessment of medication appropriateness by providing a complete and accurate overview of the patient’s current medication use.

For vulnerable patients, such as elderly patients with CKD, the first 24 h following hospital admission could be particularly important for managing medication-related problems and patient safety.

## Figures and Tables

**Table 1:** Patients’ characteristics.

Characteristic	Sample (N = 100)
Age, years, median (IQR) 65–74 75–84 ≥85	76 (71–82) 41 43 16
Gender Male, n	51
Body weight, kg, median (IQR)	75 (70–85)
Body height, cm, median (IQR)	170 (162–176)
BMI (kg/m^2^), median (IQR)	26.1 (23.8–29.7)
Serum creatinine (µmol/L), median (IQR)	310.5 (180–500)
Creatinine clearance (ml/min), median (IQR)	18.9 (10.3–32.4)
CKD-EPI (ml/min/1.73 m^2^), median (IQR)	16.5 (8.7–28)
eGFR categories (KDIGO classification), n G2 Mildly decreased G3a Mild to moderately decreased G3b Moderately to severely decreased G4 Severely decreased G5 Kidney failure	6 5 12 29 48
Hospital admission, n Emergency Elective	90 10
Length of hospital stay (days), median (IQR)	10 (6–14.3)
Patients with ≥1 previous hospitalization, n	48
Number of comorbidities, median (IQR)	12 (9–15)
Most common individual diagnoses (ICD-10 classification) N18 Chronic kidney disease I10 Essential hypertension D50-D64 Anemia E87 Disorders of fluid, electrolyte and acid-base balance E11 Type 2 diabetes mellitus	100 83 64 53 45
Number of prescribed medications (BPMH), median (IQR)	9 (7–13)

Abbreviations: BMI—Body Mass Index, BPMH—Best Possible Medication History, CKD-EPI—Chronic Kidney Disease Epidemiology Collaboration, eGFR—estimated Glomerular Filtration Rate, IQR—interquartile range, KDIGO—Kidney Disease: Improving Global Outcomes.

**Table 2:** Pharmacotherapy complexity at hospital admission (BPMH).

**Pharmacotherapy complexity at admission (BPMH)** Less than five medications Polypharmacy (5–9 medications) Excessive polypharmacy (≥10 medications)	**Number of patients** 9 43 48
**Distribution of the main ATC drug groups (BPMH)** C Drugs acting on the cardiovascular system A Drugs acting on the alimentary tract and metabolism N Drugs acting on the nervous system B Drugs acting on blood and blood-forming organs M Drugs acting on the musculoskeletal system R Drugs acting on the respiratory system G Drugs acting on the genitourinary system and sex hormones H Drugs acting on the endocrine system V Various J Anti-infectives for systemic use L Antineoplastic and immunomodulating agents S Sensory organ drugs	**Number of drugs** (%) 414 (42.4) 211 (21.6) 124 (12.7) 76 (7.8) 37 (3.8) 27 (2.8) 25 (2.6) 23 (2.4) 23 (2.4) 7 (0.7) 7 (0.7) 2 (0.2)

Abbreviations: ATC—Anatomical Therapeutic Chemical, BPMH—Best Possible Medication History.

**Table 3:** Prevalence and distribution of polypharmacy, DDIs, PIMs, and inappropriately prescribed RRDs in the study population.

Variable	Patients with ≥1, n	Median (IQR) per Patient
Polypharmacy	91	9 (7–13)
PIMs	89	3 (1–4)
RRDs requiring dose adjustment	52	1 (0–1)
Contraindicated RRDs	62	1 (0–2)
Total RRDs	82	2 (1–3)
pDDIs	94	7 (4–12)

Abbreviations: pDDI—potential drug–drug interaction, PIM—potentially inappropriate medication, RRD—renal-risk drug; IQR, interquartile range.

**Table 4:** The most frequent PIMs in the BPMH.

EU(7)-PIM	ATC	N	Prevalence (N = 258) (%)
Pantoprazole	A02BC02	48	18.6
Moxonidine	C02AC05	28	10.9
Tramadol	N02AX02	27	10.5
Urapidil	C02CA06	16	6.2
Diazepam	N05BA01	14	5.4
Alprazolam	N05BA12	11	4.3
Trimetazidine	C01EB15	11	4.3
Esomeprazole	A02BC05	10	3.9
Apixaban	B01AF02	7	2.7
Vildagliptin	A10BH02	6	2.3
Pioglitazone	A10BG03	6	2.3
Ibuprofen	M01AE01	5	1.9
Metoclopramide	A03FA01	5	1.9
Sitagliptin	A10BH01	4	1.6
Rivaroxaban	B01AF01	4	1.6
Famotidine	A02BA03	4	1.6
Doxazosin	C02CA04	4	1.6

Abbreviations: ATC—Anatomical Therapeutic Chemical Classification System, EU(7)-PIM—European Union (7)-Potentially Inappropriate Medication.

**Table 5:** The most common inappropriately prescribed RRDs (≥3).

ATC	Drug	Total	Unadjusted Dose	Contraindicated Use
B01AC06	Acetylsalicylic acid	22	0	22
C02AC05	Moxonidine	20	20	0
N02AJ13	Tramadol/paracetamol	13	0	13
C08CA13	Lercanidipine	11	0	11
C09BX01	Perindopril/indapamide/amlodipine	9	0	9
C09BA04	Perindopril/indapamide	7	1	6
C01EB15	Trimetazidine	7	2	5
C09BB04	Perindopril/amlodipine	6	1	5
C03DA04	Eplerenone	5	2	3
A03FA01	Metoclopramide	5	5	0
C09BA05	Ramipril/hydrochlorothiazide	5	2	3
A10BB09	Gliclazide	4	0	4
A10BA02	Metformin	4	0	4
C09AA04	Perindopril	4	4	0
B01AF01	Rivaroxaban	4	0	4
A02BA03	Famotidine	3	3	0
C10AA07	Rosuvastatin	3	0	3
B01AE07	Dabigatran	3	0	3

Abbreviations: ATC—Anatomical Therapeutic Chemical Classification System.

**Table 6:** Potentially clinically significant drug–drug interactions classified according to Lexicomp risk category.

Drug Interaction		N	Summary
X interactions			
Doxazosin	Urapidil	2	Alpha-1 blockers may enhance the hypotensive effect of other alpha-1 blockers
Promazine	Indapamide	2	Thiazide diuretics may enhance the QTc-prolonging effect of promazine
Tamsulosin	Urapidil	2	Alpha-1 blockers may enhance the hypotensive effect of other alpha-1 blockers
Potassium citrate/hydrogen carbonate (oral)	Quetiapine	1	Drugs with significant anticholinergic effects may increase the ulcerogenic effects of potassium salts
Calcitriol	Cholecalciferol	1	Vitamin D analogs may increase the toxic effects of other vitamin D analogs
Levodopa	Metoclopramide	1	Metoclopramide may reduce the therapeutic effect of antiparkinsonian drugs (dopamine agonists)
Pramipexole	Metoclopramide	1	Metoclopramide may reduce the therapeutic effect of antiparkinsonian drugs (dopamine agonists)
Quetiapine	Ipratropium	1	Ipratropium may enhance the anticholinergic effects of quetiapine
Promazine	Furosemide	1	Loop diuretics may enhance the QTc-prolonging effect of promazine
Olanzapine	Aclidinium	1	Aclidinium may enhance the anticholinergic effects of olanzapine
D interactions (≥5)			
Bisoprolol	Moxonidine	7	Beta-blockers may enhance rebound hypertension after abrupt discontinuation of α2-agonists; α2-agonists may increase AV-block and worsen sinus node dysfunction. Clinical significance may be greater in patients with heart failure
Alprazolam	Tramadol	6	Increased risk of CNS depression
Nebivolol	Moxonidine	6	Beta-blockers may enhance rebound hypertension after abrupt discontinuation of α2-agonists; α2-agonists may increase AV-block and sinus node dysfunction. Greater clinical relevance in heart failure patients
Gliclazide	Pioglitazone	5	Thiazolidinediones may enhance the hypoglycemic effect of sulfonylureas
Moxonidine	Tramadol	5	Moxonidine may increase the risk of CNS depression
Tramadol	Quetiapine	5	Increased risk of CNS depression
C interactions (≥14)			
Bisoprolol	Furosemide	24	Loop diuretics may enhance the hypotensive effect of antihypertensives
Amlodipine	Furosemide	18	Loop diuretics may enhance the hypotensive effect of antihypertensives
Allopurinol	Furosemide	16	Loop diuretics may increase adverse effects of allopurinol and raise levels of allopurinol and oxypurinol
Furosemide	Tramadol	16	Opioids may increase adverse effects of diuretics and reduce their efficacy
Furosemide	Moxonidine	14	Loop diuretics may enhance the hypotensive effect of antihypertensives

Abbreviations: CNS—Central Nervous System, AV—atrioventricular.

## Data Availability

The data that support the findings of this study are available from the corresponding author upon reasonable request.
